# Mimical reconstruction and aesthetic repair of the nail after resection of subungual melanocytic nevus

**DOI:** 10.1186/s12893-021-01423-9

**Published:** 2021-12-20

**Authors:** Wenpeng Xu, Xiucun Li, Songhua Cao, Ning Zhang, Yong Hu

**Affiliations:** 1 Department of Hand Surgery/Foot and Ankle Surgery, The Second Hospital, Cheeloo College of Medicine, Shandong University, No.247, Beiyuan Street, Jinan, 250033 Shandong People’s Republic of China; 2 Center of Foot and Ankle Surgery of Shandong University, The Second Hospital, Cheeloo College of Medicine, Shandong University, No.247, Beiyuan Street, Jinan, 250033 Shandong People’s Republic of China

**Keywords:** Mimical reconstruction, Aesthetic repair, Subungual melanocytic nevus, Nail

## Abstract

**Background:**

This study aimed to report the outcomes of mimical reconstruction and aesthetic repair of the nail.

**Methods:**

When the pigmented bands were more than 1/2 the width of the whole nail, mimical reconstruction of the nail was performed, with a lateral toe pulp island flap covering the wound via the subcutaneous channel. If the pigmented bands were 1/4 to 2/5 the width of the entire nail, aesthetic repair of the nail was carried out by split-thickness excision under a microscope.

**Results:**

The average age of patients at the time of surgery was 14.5 years. Five patients had lesions on their toes, while three had lesions on their fingers. There were no post-operative complications. All toenails of the five patients who had undergone mimical reconstruction exhibited a well-settled flap. The nails of the three patients who underwent aesthetic repair displayed no nail malnutrition or deformity, and all nails had an aesthetic appearance.

**Conclusions:**

Both mimical reconstruction and aesthetic repair of the nail following resection of subungual melanocytic nevus are reliable and feasible. The “like tissue” repairs of complex nail defects appear to be satisfactory. All patients had excellent aesthetic outcomes.

**Level of evidence:**

V

**Supplementary Information:**

The online version contains supplementary material available at 10.1186/s12893-021-01423-9.

## Introduction

Subungual melanocytic nevi, which always appeared as melanonychia [1, 2], are caused by the proliferation of melanocytes in the nail matrix and nail bed [3], and are usually junctional nevi and rarely compound nevi [4, 5].

According to the diagnostic criteria of the subungual melanoma [2, 6] and the consensus on melanonychia nail plate dermoscopy [7], surgical resection of subungual melanocytic nevi is performed when the width of the subungual pigmented bands exceeds 3 mm. If subungual melanocytic nevi are not resected, there is a risk of malignant transformation [1, 8], as well as significant mental stress to the patient. Previous studies have shown that approximately 25–33% of cutaneous melanomas result from melanocytic nevi [8, 9], with the rate in high-risk patients, such as those with numerous nevi, potentially reaching as high as 54.2% [10]. It has been proposed that six evident lesional phases of tumor progression form the neoplastic system that affects the human epidermal melanocyte [11, 12]. However, certain findings suggested that the progression of most melanomas is far more intricate and involves different pathways, which may be influenced in part by different carcinogenic hits [13].

There is currently no consensus on the treatment of subungual melanocytic nevi with pigmented bands wider than 3 mm. Transverse elliptical matrix excision, releasing flap method, or tangential matrix excision are performed to treat subungual melanocytic nevi with a pigmented band width of 3 to 6 mm [2, 14]. Lateral longitudinal excision is carried out on subungual melanocytic nevi with pigmentation on the lateral one‑third of the nail [2]. The whole nail with pigmented subungual melanocytic nevi is resected, and the wound is then covered with skin grafts, fillet flap with the phalanx shortening, and a free flap [2, 15–17]. Importantly, these options change the post-operative nail appearance and cannot achieve aesthetically-pleasing outcomes. In this study, mimical reconstruction and aesthetic repair of the nail were performed on patients with subungual melanocytic nevi to maintain the aesthetic contour of the nail. Therefore, this study aimed to report the outcomes of mimical reconstruction and aesthetic repair of the nail.

## Patients and methods

### Inclusion and exclusion criteria

This retrospective clinical study was approved by the Research Ethical Committee of the Second Hospital of Shandong University (KYLL-2021(LW)017). Written informed consent was obtained from each patient. All methods were performed in accordance with the Declaration of Helsinki: ethical principles for medical research involving human subjects. In this study, the inclusion criteria of the patients with subungual pigmented lesions were as follows: (1) pigmented bands that were more than 3 mm or 1/4 of the whole nail width, (2) dark brown to black color, (3) more than a one-year interval from onset to surgical resection, (4) progressive increase in the width of the pigmented bands within one year, and (5) absence of pigmentation on the adjacent skin (Hutchinson’s sign). Patients with narrow pigment bands of less than 3 mm, blurred surrounding borders, nail dystrophy, and/or ulceration were excluded from this study. Hospital medical records from August 2013 to September 2020 were reviewed, with eight patients found to have undergone mimical reconstruction and aesthetic repair of the nail following nail resection, and were pathologically diagnosed with a subungual melanocytic nevus.

### Microsurgical technique

Based on the width of the pigmented bands, two different microsurgical methods were selected. When the pigmented bands were more than 1/2 the width of the whole nail, mimical reconstruction of the nail was performed. Following resection of the entire nail, the wound was covered by a lateral toe pulp island flap via a subcutaneous channel. If the width of the pigmented bands was more than 3 mm, or 1/4 to 2/5 of the whole nail, either aesthetic repair of the nail or split-thickness excision of the pigmented nail matrix and nail bed lesions under a microscope were carried out.

#### Mimical reconstruction of the nail

**Fig. 1:**
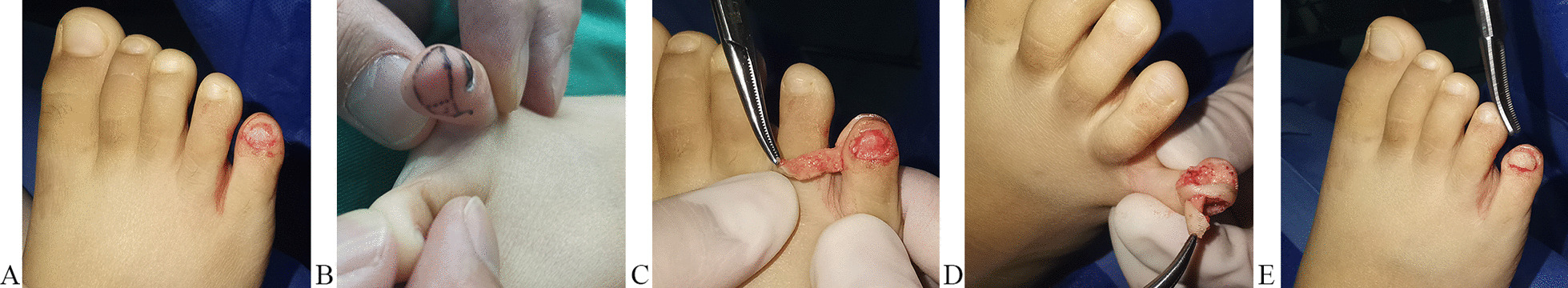
The surgical procedure of mimical reconstruction of the nail. **A** Removing the nail plate and resecting the whole nail bed and matrix. **B** Design of the lateral toe pulp island flap. **C** Dissection of the flap. **D** Transfer of the flap to the defective region of the nail bed via the subcutaneous channel. **E** The wound was covered with the flap

Mimical reconstruction of the nail is defined as reconstructing the nail using a lateral toe pulp island flap after resection of the whole nail. After removing the nail plate, the whole nail bed was resected. Furthermore, the whole nail folds were kept intact after resection of the nail bed (Fig. 1A). A lateral toe pulp island flap based on the plantar digital artery was designed according to the size of the total nail bed (Fig. 1B). A rhombic incision was made and the flap was dissected from distal to proximal, including the plantar digital nerve (Fig. 1C). The flap was transferred to the defective region of the nail bed via the subcutaneous channel (Fig. 1D) and the wound was covered (Fig. 1E). Finally, the donor site of the flap was primarily closed.

#### Aesthetic repair of the nail

**Fig. 2:**
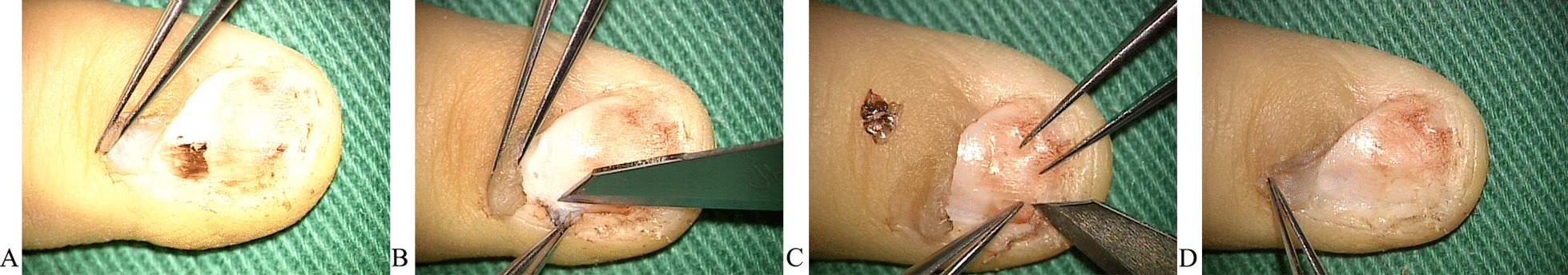
The surgical procedure of aesthetic repair of the nail. **A** Longitudinal incision. **B** and **C** Split-thickness excision procedure of the pigmented nail matrix and nail bed lesions under a microscope. **D** The residual nail matrix and nail bed were flattened under a microscope

Aesthetic repair of the nail is defined as split-thickness excision of the pigmented nail matrix and nail bed lesions under a microscope (Additional file 1: Video 1). After removing the nail plate, the origin and location of the pigmented lesions of the nail were identified. A longitudinal incision was made at the junction of the proximal nail fold and pigmented lesions (Fig. 2A). The pigmented lesions of the nail matrix and nail bed were entirely exposed. Oblique incisions were made proximally and bilaterally on the lesions. Excisional split-thickness nail matrix and nail bed were designed according to the lesion size observed under a microscope (Fig. 2B). The nail matrix and nail bed invaded by the lesion were completely removed, but the uninvaded nail matrix and nail bed were kept. (Fig. 2C). Finally, the residual nail bed was flattened under a microscope (Fig. 2D) and the incision at the proximal nail fold was sutured.

### Post-operative management

Post-operative care and monitoring were performed during the first two post-operative days. No anticoagulant was used. On the third post-operative day, patients were allowed to ambulate but were instructed to avoid any strenuous exercise for two weeks. Two weeks after surgery, the skin sutures were removed.

## Results

Among the eight patients, there were two females and six males. The average age of the patients at the time of surgery was 14.5 years (range 1 to 41 years). The mean interval from onset to surgical resection was 2.9 years (range 1 to 5 years). Five patients had lesions on their toes, while the other three had lesions on their fingers. In five out of the eight patients, the pigment bands were more than 1/2 of the nail width and these patients underwent mimical reconstruction of the nail. Aesthetic repair of the nail was carried out on the remaining three patients. Table 1 shows the basic information of the eight patients with a subungual melanocytic nevus.

**Table 1:** Basic information of the eight patients with a subungual melanocytic nevus

Patient	Sex	Age (Years)	Interval from onset to surgical resection (years)	Laterality	Site	Width of pigment bands	Colors of pigment bands	Hutchinson’s sign	Treatment
1	M	13	4	Right foot	Great toe	More than 1/2 of the whole nail width	Black	No	MR
2	M	4	4	Right foot	5th toe	The whole nail	Dark brown	No	MR
3	F	41	3	Right foot	4th toe	More than 1/2 of the whole nail width	Dark brown	No	MR
4	M	3	2	Left Foot	4th toe	The whole nail	Dark brown	No	MR
5	F	5	2	Right foot	5th toe	The whole nail	Black	No	MR
6	M	1	1	Right hand	Index finger	About 1/4 of the whole nail width	Black	No	AR
7	M	19	5	Left hand	Little finger	About 2/5 of the whole nail width	Dark brown	No	AR
8	M	30	2	Left hand	Index finger	About 1/3 of the whole nail width	Dark brown	No	AR

*M* male, *F* female, *MR* mimical reconstruction of the nail bed, *AR* aesthetic repair of the nail bed

There were no post-operative complications, such as wound infection, wound dehiscence, nail bed and/or fold necrosis, or flap necrosis. All eight patients were followed up on, with intervals ranging from 5 to 55 months (mean 19.6 months). There was no hypertrophic scar at the incision wound. The toenail of the five patients that had undergone mimical reconstruction of the nail exhibited a well-settled flap; these five patients could wear shoes and walk normally, and were satisfied with the outcomes. For the three patients that had undergone aesthetic repair of the nail, there was no nail dysrtophy, recurrence, or deformity, with all nails having an aesthetic appearance. All eight patients had satisfactory outcomes.

### Case 1 (patient 1)

**Fig. 3:**
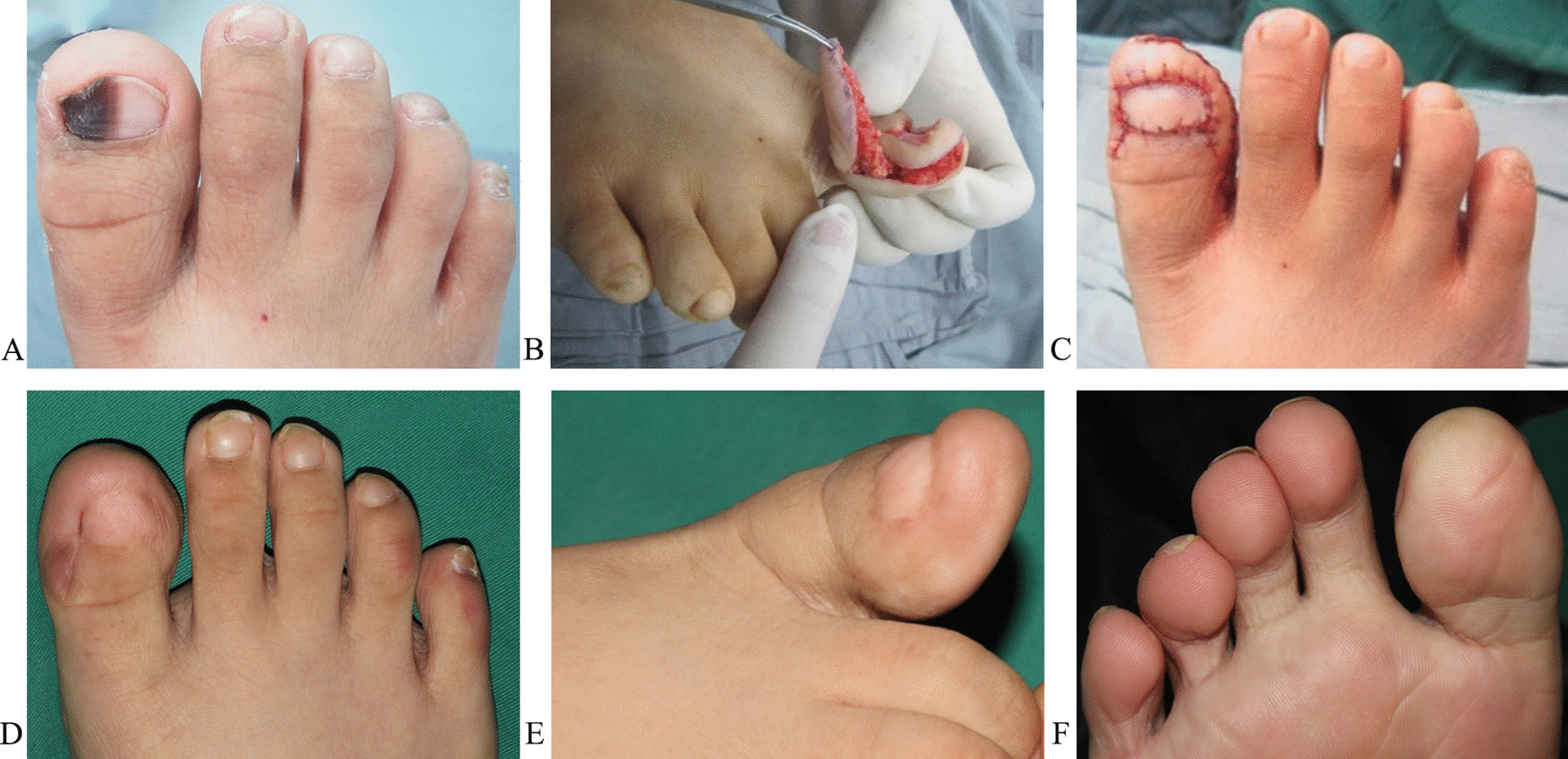
The pre-, intra-, and post-operative results of Case 1. **A** The subungual melanocytic nevus of the right hallux. **B** and** C** The defect of the nail matrix after resection of the tumor was repaired with a lateral toe pulp island flap based on the plantar digital artery. **D**–**F** The results from 16 months after surgery

A 13-year-old male patient had a history of progressive subungual melanosis in the hallux of the right foot for over 4 years, but no pseudo-Hutchinson signs (Fig. 3A). After resection, the tumor was pathologically diagnosed as a subungual melanocytic nevus. The defect of the nail matrix was repaired with a lateral toe pulp island flap based on the plantar digital artery (Fig. 3B and C). The patient was monitored for 16 months and made a full recovery after surgery (Fig. 3D–F).

### Case 2 (patient 5)

**Fig. 4:**
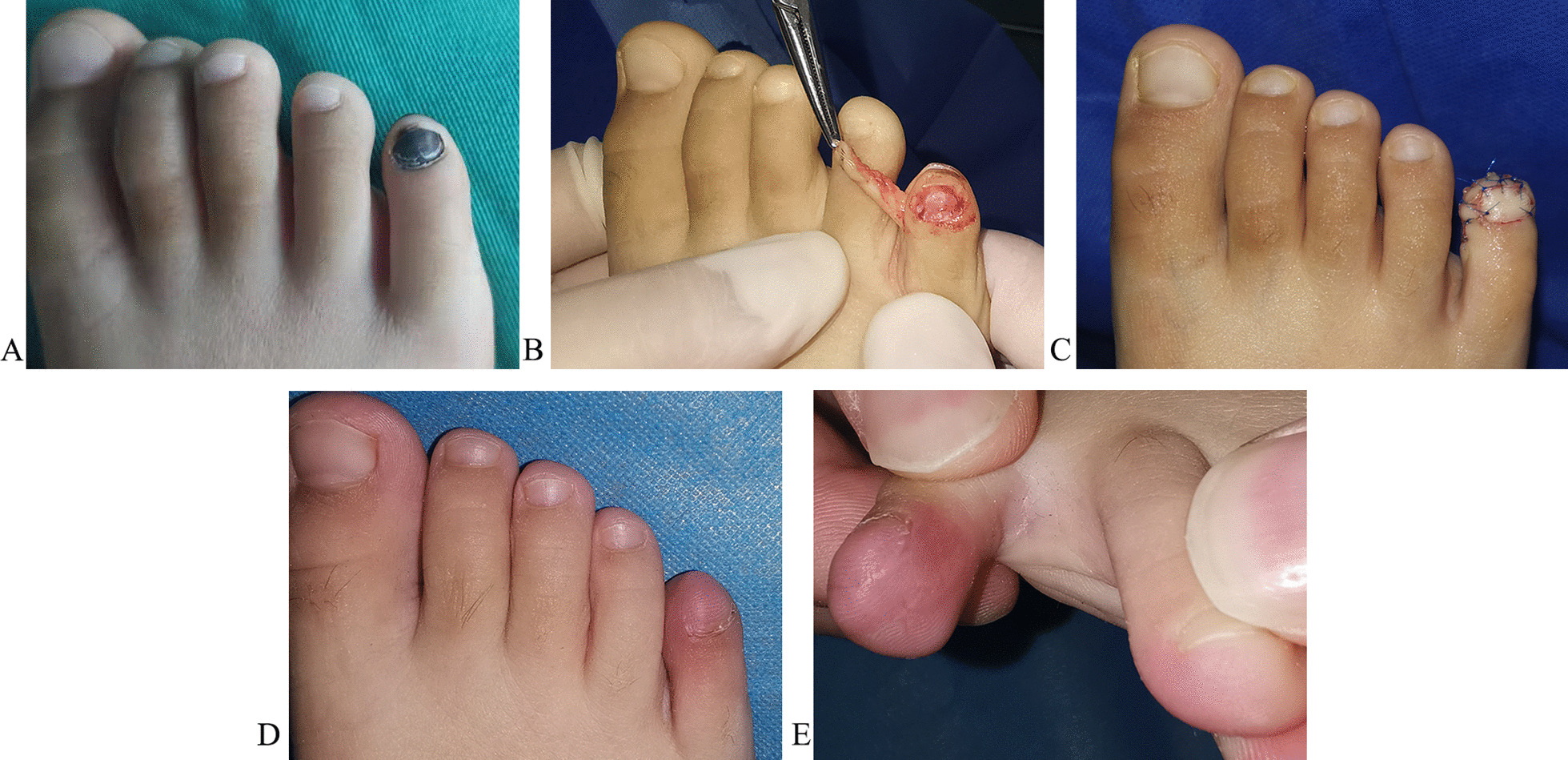
The pre-, intra-, and post-operative results of case 2. **A** The subungual melanocytic nevus of the fifth toe of the right foot. **B** and** C** The defect of the nail matrix after the resection of the tumor was repaired with a lateral toe pulp island flap based on the plantar digital artery. **D** and** E** The results from five months after surgery

A 5-year-old female patient had a history of progressive subungual melanosis for more than 2 years and intermittent pain in the fifth toe of her right foot for more than half a year (Fig. 4A). After resection, the tumor (total nail matrix) was pathologically diagnosed as a subungual melanocytic nevus of the fifth toe. The defect of the total nail matrix was repaired with a lateral toe pulp island flap based on the plantar digital artery (Fig. 4B and C). The patient was monitored for five months and had a satisfactory outcome (Fig. 4D and E).

### Case 3 (patient 6)

**Fig. 5:**
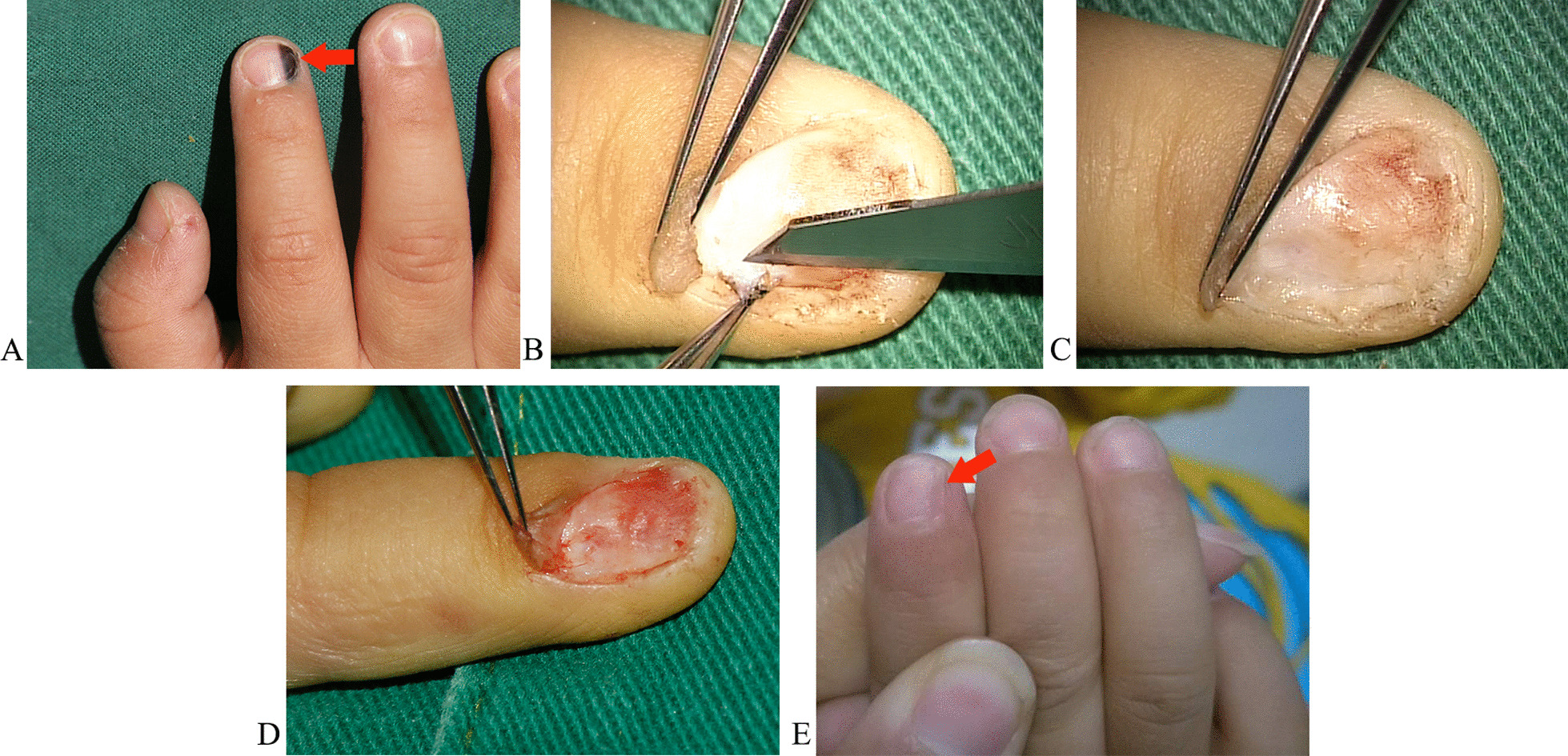
The pre-, intra-, and post-operative results of Case 3. **A** The subungual melanocytic nevus on the index finger of the right hand. **B** and** C** Split-thickness excision of the pigmented nail bed lesions under a microscope. **D** The residual nail bed was flattened. **E** The result from 20 months after surgery

A 1-year-old boy was pathologically diagnosed with a subungual melanocytic nevus on his right index finger (Fig. 5A). The patient had been suffering from progressive subungual melanosis for over half a year. After removing the nail plate, split-thickness excision of the pigmented nail bed lesions was performed under a microscope (Fig. 5B and C). Furthermore, the residual nail bed was flattened under a microscope (Fig. 5D). The patient was monitored for 20 months and had a satisfactory outcome (Fig. 5E).

## Discussion

In this study, mimical reconstruction and aesthetic repair of the nail following resection of subungual melanocytic nevus were reliable and feasible, with all patients achieving satisfactory appearances.

Although melanocytes are the normal pigment-producing cells in the skin [1], their density in the nail matrix (200/mm^2^) and nail bed (absent to 50/mm^2^) is much smaller than in normal skin (1150/mm^2^) [3, 5]. Some subungual melanocytic nevi are not black, thus evaluating the malignant degree of pigmented lesions based solely on color is not reliable.

The thickness of a normal nail bed in healthy individuals is approximately 1.17 mm [18]. Melanocytes in the nail matrix are usually found in the suprabasal position between the second and fourth layers; in the nail bed, all melanocytes are found in the first and second layers [3, 5, 19]. Di  Chiacchio et al. reported that the average thickness (depth) of subungual pigmented lesions is 0.08 mm (range 0.04 to 0.12 mm), while the thickness of subungual melanocytic nevi ranged from 0.04 to 0.07 mm (mean: 0.05 mm) [20]. In our study, five patients had pigmented bands varying in width from 1/4 to 2/5 of the whole nail. These five patients underwent split-thickness excision of the pigmented nail matrix and nail bed lesions under a microscope for aesthetic repair. There was no tumor recurrence in the patients after more than 1 year. Therefore, split-thickness excision of the pigmented nail matrix and nail bed lesions under a microscope is reliable and feasible for aesthetic repair. When the pigmented bands were more than 1/2 the width of the whole nail, split-thickness excision of the pigmented nail matrix and nail bed lesions under a microscope may lead to nail malnutrition or deformity. Thus, whole nail resection is an option.

The absence of a nail may not only affect the appearance and function of the injured foot/hand but also place a psychological burden on patients due to the poor appearance of the toe/finger. In addition to improving the sensitivity and stability of the toe pulp, the nail has an aesthetic effect [21–23]. Even now, significant attention continues to be placed on the aesthetic reconstruction of fingernail defects [21, 23, 24], whereas emulational repair and aesthetic reconstruction of the toenail defects have long been neglected. Aesthetic reconstruction of toenail soft tissue defects has become increasingly important as aesthetic knowledge increases. Therefore, reconstruction of the toenail soft tissue defects poses a major challenge for surgeons.

Wound repair following resection of a whole nail is also a brainteaser. In clinical practice, skin grafts used to repair soft tissue defects of the nail with phalanx exposure often results in a hypertrophic scar at the recipient site [16]. A fillet flap with phalanx shortening often requires sacrificing the distal phalanx [15]. The disadvantages of a free flap include long operative time and microsurgical microvascular anastomosis [17]. Currently, few studies focus on using toe pulp flaps for reconstructing nail defects. Cheng et al. used a lateral toe pulp flap to repair the dorsal toe defect [25] while Tashiro et al. used a second-toe lateral hemipulp flap transfer to cover a third-toe pulp defect [26]. In our study, mimical reconstruction of the nail following resection of the whole nail was performed using a lateral toe pulp island flap. All patients were satisfied with their excellent functional and aesthetic outcomes. There are several advantages of using a lateral toe pulp island flap over conventional flaps, including a shorter operating time, simple flap dissection, minimal donor site morbidities, and outstanding functional and aesthetic outcomes [15–17]. In addition, the texture and color of the toe pulp are similar to that of the toenail because it has a thicker horny layer. This flap can mimic the unique original characteristics of the toenail to minimize deformities. Furthermore, the toe pulp can provide a glabrous skin flap suitable for resurfacing toenail soft tissue defects, allowing for sensate reconstruction by “replacing like with like”.

## Conclusions

Mimical reconstruction and aesthetic repair of the nail following resection of subungual melanocytic nevus are reliable and feasible. Complex nail defects repaired by “like tissue” appear to be satisfactory. All patients had excellent aesthetic outcomes.

## Supplementary Information

**Additional file 1.** The surgical procedure of aesthetic repair of the nail under a microscope.

## Data Availability

The datasets used and/or analysed during the current study available from the corresponding author on reasonable request.

## Abbreviations

MR: Mimical reconstruction of the nail bed
AR: Aesthetic repair of the nail bed
